# Peripheral Inflammatory Biomarkers in Chronic Autoimmune-Associated Epilepsy: An Exploratory Case–Control Study

**DOI:** 10.3390/biom16091350

**Published:** 2026-09-16

**Authors:** Laura Serrano-Aguilar, Cristina Fernandez-Hombrados, Mercedes Jimenez-Vargas, Yolanda López-Moreno, Guillermina García-Martín, Pablo Cabezudo-García, Nicolas Lundahl Ciano-Petersen, Guillermo Estivill-Torrús, Manuel Narváez-Peláez, Maria José Aguilar-Castillo, Virgilio Hernándo-Requejo, Pedro Jesus Serrano-Castro

**Affiliations:** 1 Faculty of Medicine, San Pablo CEU University, Av. de Montepríncipe, 28668 Alcorcón, Spain; laura.serranoaguilar@gmail.com (L.S.-A.); cristina.fhombrados@gmail.com (C.F.-H.); vhernando@ext.hmhospitales.com (V.H.-R.); 2 Neuroscience Unit, Neurology Service, University Regional Hospital of Málaga, 29010 Málaga, Spain; yolandalopm@gmail.com (Y.L.-M.); guillerminagmartin@gmail.com (G.G.-M.); pablocabezudo@gmail.com (P.C.-G.); 3 Malaga Biomedical Research Institute and Nanomedicine Platform (IBIMA Plataform BIONAND), 29590 Málaga, Spain; nico.lundahl@ibima.eu (N.L.C.-P.); guillermo.estivill@ibima.eu (G.E.-T.); maijo.aguilarcastillo@gmail.com (M.J.A.-C.); 4 Red Andaluza de Investigación Clínica en Neurologia (Neuro-RECA), 29010 Málaga, Spain; mnarvaez@uma.es; 5 Department of Physiology, University of Malaga, 29010 Málaga, Spain; 6 Laboratory Service, Regional University Hospital of Málaga, 29010 Málaga, Spain; 7 Neurology Service, La Paz University Hospital, P.º de la Castellana, 261, Fuencarral-El Pardo, 28046 Madrid, Spain; 8 Department of Medicine and Dermatology, University of Malaga, 29010 Málaga, Spain

**Keywords:** autoimmune associated epilepsy, neuroinflammation, biomarkers, IL-12, epilepsy

## Abstract

Background: Autoimmune-associated epilepsy (AAE) was recognized by the ILAE in 2017 as a distinct etiological category driven by chronic neuroinflammation rather than structural or genetic mechanisms. Reliable peripheral circulating inflammatory biomarkers to distinguish AAE from structural epilepsy remain lacking, potentially limiting timely recognition and evaluation for immunotherapy. In this observational case–control exploratory single-center study, plasma levels of 20 pro-inflammatory and 3 anti-inflammatory biomarkers, plus cerebrospinal fluid neurofilament light chain and HMGB1, were measured in 18 AAE patients, 33 non-autoimmune epilepsy patients and 16 healthy controls. AAE was defined using Dubey’s APE2-based criteria. Univariate comparisons, ROC analysis, and binary logistic regression were performed. Results: Among the 25 biomarkers analyzed, plasma IL-12 showed the lowest nominal *p*-value in the global between-group comparison (p=0.038), with lower concentrations in AAE. However, no biomarker remained statistically significant after Benjamini–Hochberg false discovery rate correction (IL-12 q=0.366). ROC analysis yielded an AUC of 0.670 for IL-12 alone for distinguishing AAE from the remainder of the cohort. In an exploratory primary multivariable model including plasma IL-12, drug-resistant epilepsy (DRE) status, and EEG pattern, the apparent in-sample discriminative performance was higher than that of IL-12 alone (AUC = 0.771; 95% CI, 0.635–0.892). Discussion: Lower plasma IL-12 levels may reflect a peripheral immune association in chronic AAE. These findings are exploratory and hypothesis-generating and require replication in larger, prospectively recruited, independent cohorts.

## 1. Introduction

Traditionally epilepsy has been attributed to structural causes, associated with identifiable brain lesions, or to genetic factors, linked to increased individual susceptibility to epileptic seizures. However, a substantial proportion of epileptic syndromes did not fit within these conventional categories [1,2]. In recent years, relevant evidence has accumulated on new etiologies involved in epilepsy. In this context, the 2017 International League Against Epilepsy (ILAE) classification of epilepsies recognized immune etiology as an independent category, defined by inflammation of the central nervous system of autoimmune origin, in which epilepsy constitutes a direct manifestation of immune dysfunction [2]. Autoimmune epilepsies are characterized by autoantibodies against neuronal antigens, either cell-surface or intracellular, and/or by active neuroinflammatory processes, sometimes associated with specific biomarkers, that directly influence neuronal excitability [3,4,5,6].

Immune-associated epilepsies can be classified according to the main pathophysiological mechanisms involved. One group includes epilepsies mediated by antibodies against synaptic receptors and membrane complexes, such as anti-NMDAR, anti-AMPAR, anti-GABA-A, anti-GABA-B, anti-LGI1, anti-CASPR2, and anti-mGluR5. These antibodies alter the density and function of synaptic receptors and ionotropic channels, thereby modifying neuronal excitability, usually without causing extensive neuronal destruction [7,8,9,10]. A second group comprises chronic epilepsies associated with antibodies against intracellular antigens, including anti-GAD65, anti-Hu, and other onconeural antibodies. These epilepsies are more closely linked to cytotoxic T-cell–mediated processes and irreversible neuronal damage, particularly in paraneoplastic contexts, and typically show a limited response to immunotherapy [11]. A third group includes epilepsies with active neuroinflammation but no identified autoantibodies. These conditions are characterized by microglial activation, peripheral immune-cell infiltration, and pro-inflammatory cytokine production. This group includes epilepsies in which imaging, CSF, or histological findings support neuroinflammation, such as Rasmussen encephalitis or some drug-resistant chronic epilepsies [12]. Across these groups, evidence consistently indicates blood–brain barrier dysfunction, which facilitates the entry of autoantibodies and immune cells into the central nervous system [13].

The third subgroup is particularly relevant because, unlike the first two groups, it lacks direct biomarkers such as autoantibodies, making it more difficult to identify. Histopathological studies of autoimmune epilepsies have shown inflammatory infiltrates containing cytotoxic T lymphocytes and B cells, along with microglial and astrocytic activation, and probable dysregulation of the neuroinflammatory balance [14,15,16].

In contrast, structural epilepsies arise from anatomical abnormalities detectable on structural neuroimaging [1]. Although inflammatory mechanisms may contribute to epileptogenesis in these types of epilepsies [17,18], the primary pathophysiology of structural epilepsies is driven by anatomical alterations that generate epileptogenic foci through mechanisms distinct from active autoimmune neuroinflammation.

From a clinical point of view, among immune-related epileptic disorders, two entities should be distinguished. The first comprises acute symptomatic seizures secondary to immune-mediated limbic encephalitis. These episodes do not constitute epilepsy per se, as seizure recurrence is not expected once the acute condition has been resolved. They are usually associated with autoantibodies against neuronal surface antigens, which may be directly epileptogenic, and their medium- to long-term prognosis after the acute phase is typically favorable [4]. The second entity corresponds to autoimmunity-associated epilepsy (AAE), a chronic form driven by a persistent neuroinflammatory pathogenic process. AAE is often associated with antibodies against intracellular antigens, such as anti-GAD65 or onconeural antibodies, although identifiable biomarkers may be absent. These epilepsies are usually drug-resistant [4]. Both AAE and acute symptomatic seizures related to limbic encephalitis may respond to immunotherapy, including corticosteroids, immunoglobulins, or rituximab, in combination with antiseizure medication (ASM) [19,20], while in the case of structural epilepsies, treatment is mainly based on ASM, with consideration of non-pharmacological options (surgery or neuromodulation) when they are drug-resistant. This distinction may become relevant, given that some cases of AAE have been shown to have reversibility potential with appropriate immunological treatment, especially when associated with neuronal surface antibodies [21].

Studies of peripheral circulating inflammatory biomarkers in epilepsy have shown alterations in both plasma and cerebrospinal fluid (CSF). Among the peripheral circulating inflammatory biomarkers most frequently examined in the literature are cytokines [22], which are immune-cell–derived molecules that modulate inflammatory responses by either promoting or suppressing them. The main pro-inflammatory cytokines include TNF-α, IL1-β, and IL-6, as well as other interleukins, such as IL-8, IL-12, and IL-18, selected interferons—particularly IFNγ—and factors such as GM-CSF, which together coordinate the systemic inflammatory response. Key anti-inflammatory cytokines include IL-10 and IL-4. Chemokines constitute a cytokine subfamily with specific roles in leukocyte migration and are secreted by cells such as T lymphocytes and astrocytes. Particularly relevant examples include MCP-1 (monocyte chemoattractant protein-1), also known as CCL2, and fractalkine (CX3CL1), whose soluble form contributes to communication between neurons and inflammatory cells [23].

Beyond cytokines, HMGB1/TLR-4, ICAM-1/selectins, and NfL reflect neuroinflammation-related microglial activation, leukocyte recruitment, and axonal injury, supporting their potential as peripheral circulating inflammatory biomarkers of epileptogenesis [24]. Nevertheless, despite recent advances [3,22], important gaps remain in defining the precise peripheral inflammatory profile of this type of epilepsy, largely because most studies do not clearly distinguish between etiological subtypes and differ in biomarker assessment methods, inclusion criteria, diagnostic definitions, and translation into clinically applicable tools.

These limitations underscore the need for molecular biomarkers to detect AAE regardless of autoantibody status. Distinct inflammatory profiles, including NfL and cytokine changes, support characteristic molecular signatures [3,25]. Differential biomarkers may accelerate autoimmune epilepsy diagnosis, guide early treatment, monitor immunotherapy response, and support targeted therapies. This study uses a well-characterized cohort and validated methods to identify neuroinflammatory profiles distinguishing autoimmune epilepsy from structural epilepsy and healthy controls.

The objective of this exploratory study was to assess whether a panel of circulating inflammatory biomarkers differs among patients with chronic AAE, patients with non-autoimmune-related epilepsy (NAE), and healthy controls (HC).

## 2. Materials and Methods

### 2.1. Study Design

This was an observational, analytical case–control study conducted at the Epilepsy Unit of the Regional University Hospital of Málaga, Spain.

### 2.2. Study Population

#### Inclusion and Exclusion Criteria

HC group:

- Inclusion criteria: Individuals without a diagnosis of epilepsy, other neurological disorders, chronic inflammatory diseases, autoimmune conditions, or active infectious diseases.
- Exclusion criteria: First-degree family history of epilepsy; any clinically relevant condition that could alter neuroinflammatory biomarkers; or chronic use of immunomodulatory or anti-inflammatory medications.

NAE group:

- Inclusion criteria: Patients with epilepsy diagnosed according to International League Against Epilepsy (ILAE) criteria who were classified as having either structural epilepsy, based on a structural lesion on brain MRI considered the most likely epileptogenic substrate, or non-lesional epilepsy not fulfilling diagnostic criteria for AAE. Eligible lesions included hippocampal sclerosis, focal cortical dysplasia, gliosis or post-traumatic/post-surgical scarring, low-grade glial tumors, cortical developmental malformations, or non-lesional epilepsy not fulfilling diagnostic criteria for AAE.
- Exclusion criteria: Evidence of current or previous autoimmune encephalitis; presence of neuronal autoantibodies; active progressive epileptic syndromes of genetic or immunological origin; or significant neurological or inflammatory comorbidities unrelated to structural epilepsy.

AAE Group:

- Inclusion criteria: Patients fulfilling the criteria proposed by Dubey et al. [26] for autoimmune-associated epilepsy, classified as:
  ○ Definite autoimmune epilepsy: Epilepsy of unknown etiology with clinically validated positive neuronal autoantibodies. Antibody-positive cases were classified as definite AAE only when antibody results were considered clinically concordant with the seizure phenotype and ancillary investigations by the multidisciplinary epilepsy/neuroimmunology team.
  ○ Possible autoimmune epilepsy: Epilepsy of unknown etiology with an Antibody Prevalence in Epilepsy and Encephalopathy (APE2) score ≥ 4 and negative neuronal autoantibodies. The APE2 score was calculated according to the presence of the following features: rapid onset of seizures or de novo altered mental status (+1); neuropsychiatric symptoms (+1); autonomic dysfunction (+1); viral prodrome (+2); faciobrachial dystonic seizures (+3); facial dyskinesias (+2); seizures refractory to at least two antiseizure medications (+2); inflammatory cerebrospinal fluid profile (+2); MRI findings suggestive of encephalitis (+2); and presence of systemic cancer (+2).
- Exclusion criteria: Evidence of active encephalopathy not related to autoimmune mechanisms and significant neurological or inflammatory comorbidities unrelated to autoimmune epilepsy.

Because circulating cytokines are influenced by systemic conditions, clinical records were reviewed for relevant inflammatory and metabolic comorbidities, recent infection, active malignancy, immunomodulatory treatment, and concomitant medication. Participants with active infection or clinically significant inflammatory disease unrelated to epilepsy were excluded whenever identified. However, data on all potential determinants of circulating cytokines, including menopausal status, adiposity, smoking, and subclinical metabolic disease, were not available for all participants.

### 2.3. Sample Size

The study included 51 patients with epilepsy and 16 healthy controls. Patients were classified by etiology as having AAE or NAE. This was an exploratory study based on a clinically well-characterized, previously recruited cohort. Because there were no robust prior data allowing estimation of expected effect sizes for the selected plasma biomarker panel in chronic AAE, no formal a priori sample-size calculation was performed. The sample size was therefore determined by cohort availability. We prespecified that all findings would be interpreted as exploratory and intended to inform the design of future adequately powered validation studies.

### 2.4. Variables

We analyzed demographic and clinical variables, together with neuroinflammatory biomarkers, including:

#### 2.4.1. Demographic and Clinical Variables

Age, sex, age of onset of epilepsy, duration of epilepsy, type of seizures, frequency of seizures, number and type of ASMs, brain MRI findings, electroencephalographic findings, specific etiology, APE2 score. In AAE group: type of antibodies, response to immunotherapy.

#### 2.4.2. Biomarkers of Neuroinflammation

○ Plasma pro-inflammatory biomarkers (20): MIP-1α (CCL3), IL-1β, IP-10 (CXCL10), IL-8 (CXCL8), IL-12, IL-17 α, IL-33, IFN-γ, GM-CSF, TNF-α, MIP-1β, IFN-α, MCP-1, P-selectin (CD62P), IL-1α, ICAM-1, E-selectin (CD62E), sTNF-RII, TLR4, HMGB1.
○ CSF pro-inflammatory biomarkers (1): HMGB1.
○ Plasma anti-inflammatory biomarkers (3): IL-4, IL-10, IL-13.
○ Biomarker of neuronal damage in CSF (1): Neurofilaments light chain (NfL).
○ Specific autoantibodies.

Biomarkers were selected a priori to represent complementary inflammatory and injury-related pathways implicated in epileptogenesis, including innate and adaptive cytokine signaling, chemotaxis, endothelial activation, damage-associated molecular patterns, soluble TNF-receptor signaling, and axonal injury. Selection was informed by a prior systematic review of neuroinflammation biomarkers in epilepsy, previous work from our group [18], and the availability of a standardized multiplex inflammation panel. The multiplex panel was complemented by targeted ELISA measurements of HMGB1, TLR4, sTNF-RII, and CSF NfL.

### 2.5. Sample Collection and Processing

#### 2.5.1. Sample Collection

Samples were obtained from a previously recruited and clinically characterized cohort. Blood and CSF samples were collected and processed as follows:

- Plasma: Venous blood collection in tubes with sodium citrate, centrifugation at 2500× *g* for 10 min at room temperature, storage at −80 °C
- CSF: Standard lumbar puncture, centrifugation at 900× *g* for 10 min, aliquot and storage at −80 °C

Peripheral blood was collected into sodium citrate tubes. Plasma was selected for this study because it was the matrix available within the standardized biobanking workflow and is compatible with the multiplex and ELISA platforms used. Samples were centrifuged at 2500× *g* for 10 min at room temperature, aliquoted, and stored at −80 °C until batch analysis. All reported peripheral biomarker concentrations therefore refer to citrate plasma, not serum. Use of citrate plasma improves internal consistency because the same matrix was used across all participants, but it limits direct comparison with studies measuring serum biomarkers or using EDTA/heparin plasma.

For each participant, we recorded date and clock time of venipuncture, fasting status, current treatment, and interval from the last clinically recognized seizure. Samples were obtained between 10:00 and 12:00 h, under fasting conditions, during routine outpatient assessment for both patients and healthy controls. No cortisol measurements were available.

#### 2.5.2. Determination of Biomarkers

- Inflammatory Biomarkers: ProcartaPlex™ Human Inflammation Panel, 20 plex (Thermo Fisher Scientific, Waltham, MA, USA) with xMAP^®^ technology on Luminex MAGPIX^®^ equipment.
- HMGB1 and TLR4: Kits ELISA ElabScience^®^ (Elabscience, Houston, TX, USA).
- sTNF-RII: Kit ELISA Human sTNF RII/TNFRSF1B Quantikine^®^ (R&D Systems, Minneapolis, MN, USA).
- NfL in CSF: Kit ELISA UmanDiagnostics NF-light™ (Quanterix Corporation, Billerica, MA, USA).

#### 2.5.3. Determination of Autoantibodies

Neuronal autoantibodies were detected by indirect immunofluorescence (IIFT) in transfected cells, using a cell-based assay (CBA) with BIOCHIP Mosaics (IIFT Autoimmune Encephalitis Mosaic 6, EUROIMMUN), against the following surface antigens: contact-associated protein type 2 (CASPR2), dipeptidyl-peptidase-like protein 6 (DPPX), leucine-rich inactivated protein 1 (LGI1), N-methyl-D-aspartate receptor (NMDAR), GABA receptor (GABABR) and α-amino-3-hydroxy-5-methyl-4-isoxazolepropionic acid receptor (AMPAR).

Immunoblotting (EUROLINE, kit for paraneoplastic neurological syndromes of 12 antigens, EUROIMMUN), including amphiphysin, recoverin, titin, Zic4, SOX1, CV2/CRMP5, paraneoplastic antigen Ma2 (PNMA2, Ma2/Ta), Ri (ANNA-2), Hu (ANNA-1), Yo (PCA-1), the 65 kDa isoform of glutamic acid decarboxylase (GAD65) and Tr (DNER) was used for the determination of antibodies directed against intracellular antigens.

Positive or clinically discordant results were evaluated using confirmatory tissue-based indirect immunofluorescence and/or in-house cell-based assays, as appropriate. Detailed confirmatory procedures are provided in Supplementary Section S1, and a representative histoimmunochemical image is provided in Figure S1.

The detection of anti-GAD65 antibodies, both by immunoblot and after confirmation by IIFT/CBA, was considered clinically significant when titers were elevated (>1:3000) [27].

### 2.6. Statistical Analysis

Descriptive analyses included means and standard deviations (SDs) for continuous variables, and frequencies and percentages for categorical variables. The Shapiro–Wilk test was used to assess distribution normality.

Univariate analyses were then performed to compare biomarker concentrations across the three groups. Normally distributed variables were analyzed using ANOVA followed by Bonferroni post hoc tests, whereas non-normally distributed variables were analyzed using the Kruskal–Wallis test followed by Dunn’s post hoc tests with Bonferroni correction. Categorical variables were compared using the chi-square test or Fisher’s exact test, as appropriate.

Given the exploratory analysis of multiple plasma biomarkers, *p*-values from global between-group comparisons were additionally adjusted using the Benjamini–Hochberg procedure to control the false discovery rate. Adjusted *p*-values are reported as q-values. Nominal *p*-values are presented for descriptive and hypothesis-generating purposes.

ROC curves were generated for biomarkers showing nominal between-group differences in the exploratory univariable analyses. The area under the curve (AUC) was calculated with 95% confidence intervals, optimal cut-off points were determined using the Youden index, and sensitivity and specificity were estimated.

For the exploratory multivariable analysis, only participants with epilepsy were included. Binary logistic regression was used to distinguish AAE from NAE. HC were excluded because epilepsy-specific clinical and EEG variables were not applicable to this group.

All statistical analyses were performed using IBM SPSS Statistics for Windows, version 25.0 (IBM Corp., Armonk, NY, USA).

### 2.7. Ethical Considerations

The study was conducted in accordance with the Declaration of Helsinki and approved by the Research Ethics Committee of Málaga. Written informed consent was obtained from all participants or their legal representatives before inclusion, using the attached consent form.

Samples and clinical data were stored in encrypted form to ensure participant de-identification. Access was limited to authorized members of the research team, in compliance with the European General Data Protection Regulation.

This study forms part of the project “Personalized medicine in neurological diseases through the application of biomarkers for the improvement of the diagnosis, prognosis and treatment of the patient. Program for diseases associated with neuroglial antibodies”, which was approved by the Biomedical Research Ethics Committee of Andalusia (Protocol Code: 1228-N-23) on 23 June 2023.

## 3. Results

### 3.1. Descriptive Analysis

Patients with epilepsy were consecutively enrolled at the Epilepsy Unit of the Regional University Hospital of Málaga. Blood and CSF samples from patients with epilepsy were collected between March 2019 and August 2021. HC blood samples were collected between April 2019 and October 2023. Overall, biological samples were collected between March 2019 and October 2023. Laboratory determinations were performed between March 2019 and October 2023.

A cohort of 67 participants was recruited and initially divided into four groups: AAE (*n* = 18), StrE (*n* = 11), non-lesional epilepsy (NLE; *n* = 22), and HC (*n* = 16). Among the 18 patients classified as AAE, 14 were antibody-positive and 4 were seronegative. For the primary biomarker analyses, the StrE and NLE subgroups were combined into the NAE group.

Participant flow, cohort allocation, sample availability, and the handling of missing data are summarized in Supplementary Figure S2.

The groups showed similarity in the initial demographic and clinical parameters, such as age, sex, onset of epileptic syndrome, duration of epilepsy, number of concomitant ASMs and mechanisms of action of the ASMs used. However, statistically significant differences were observed in seizure type, EEG findings, etiology, refractoriness, seizure frequency, and APE2 score (Table 1).

The clinical and demographic details of the AAE group are summarized in Table 2.

### 3.2. Univariate Analysis of Biomarkers

A univariate analysis was conducted to compare 22 plasma molecules and 3 CSF molecules across three study groups: HC, AAE, and NAE, which included the previously defined NLE and StrE categories. Descriptive results, expressed as mean (±SD) and median (IQR), together with statistical significance values, are presented in Table 3. Group comparisons were performed using ANOVA for normally distributed variables and the Kruskal–Wallis test for non-normally distributed variables.

**Figure 1:**
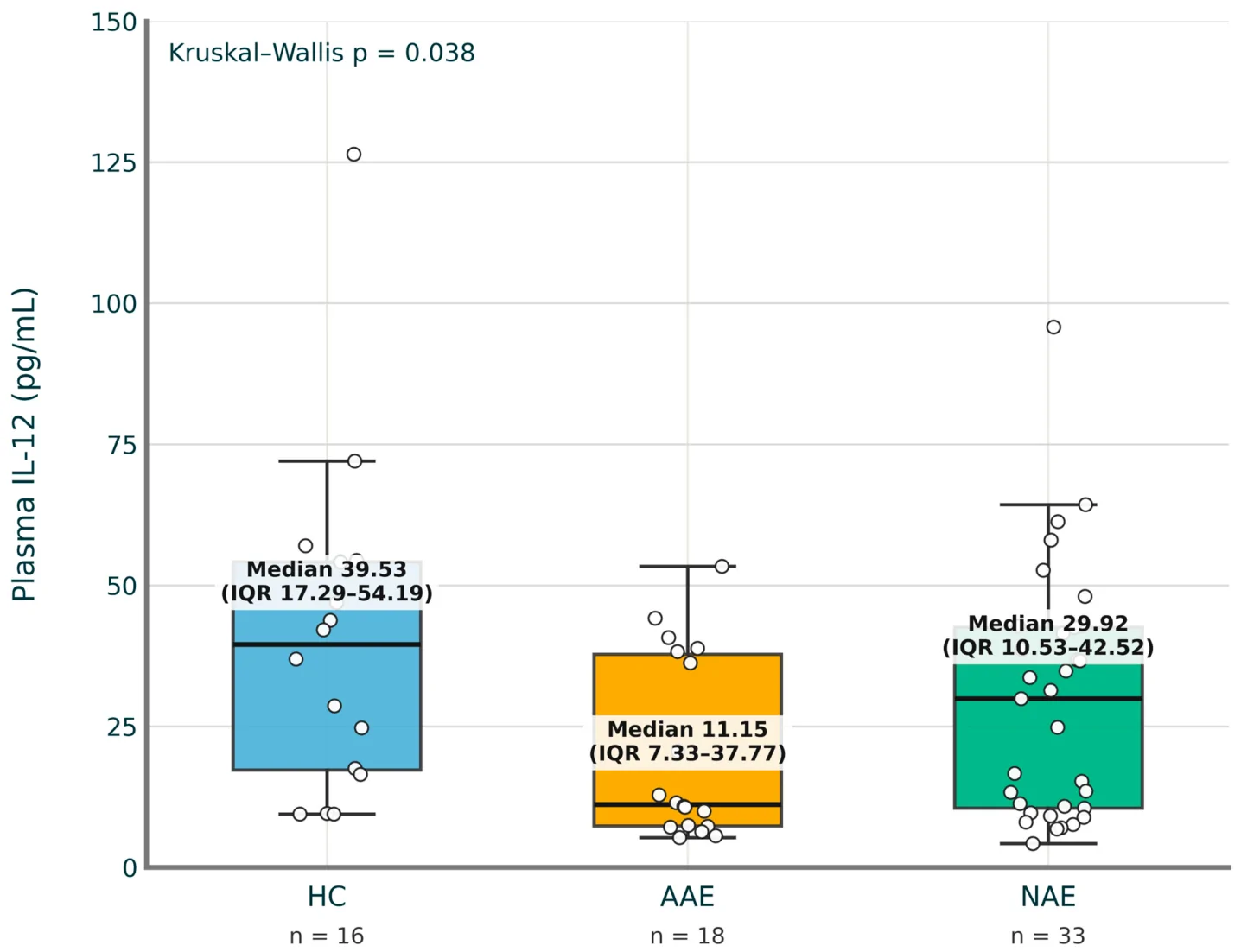
Plasma IL-12 concentrations by study group. IL-12 showed a nominal global between-group difference (*p* = 0.038); this finding did not remain significant after Benjamini–Hochberg false discovery rate correction (*q* = 0.366). White circles represent individual participant values.; central lines and boxes represent median and IQR.

No biomarker remained statistically significant after Benjamini–Hochberg false discovery rate correction. Plasma IL-12 showed the lowest nominal *p*-value in the global comparison across groups (*p* = 0.038; *q* = 0.366). Figure 1 shows the comparison of median IL-12 levels by study group.

### 3.3. Multivariate Analysis

Because plasma IL-12 was the only biomarker showing a nominal between-group difference in the univariate analysis, an exploratory binary logistic regression model was fitted among participants with epilepsy. Plasma IL-12 concentration, DRE status, and multifocal IEDs were included as candidate predictors of AAE versus NAE (Table 4).

This exploratory binary logistic regression model was fitted to distinguish AAE (*n* = 18) from NAE (*n* = 33). The model showed a significant overall fit (likelihood-ratio $\chi^{2}=12.05$, 3 degrees of freedom, *p* = 0.007), with a Nagelkerke’s $R^{2}$ of 0.289 and an apparent in-sample classification accuracy of 70.6%. In this exploratory model, higher plasma IL-12 concentrations were nominally associated with lower odds of AAE (OR = 0.964 per pg/mL; 95% CI, 0.929–1.000; *p* = 0.048). DRE was independently associated with higher odds of AAE (OR = 13.17; 95% CI, 1.45–119.61; *p* = 0.022), whereas multifocal IEDs were not independently associated with AAE status (OR = 1.76; 95% CI, 0.38–8.19; *p* = 0.473). The association between lower plasma IL-12 concentration and AAE classification remained nominally significant in a parsimonious exploratory model; the estimate is imprecise and requires external validation.

An alternative exploratory model including APE2 score is reported in Table S1. Because APE2 contributed to the operational definition of seronegative AAE, this analysis was considered descriptive and was not interpreted as an independent diagnostic prediction model.

The direction of association observed across the exploratory models should be considered hypothesis-generating rather than confirmatory evidence of discrimination between AAE and NAE.

### 3.4. Generation of ROC Curves

**Figure 2:**
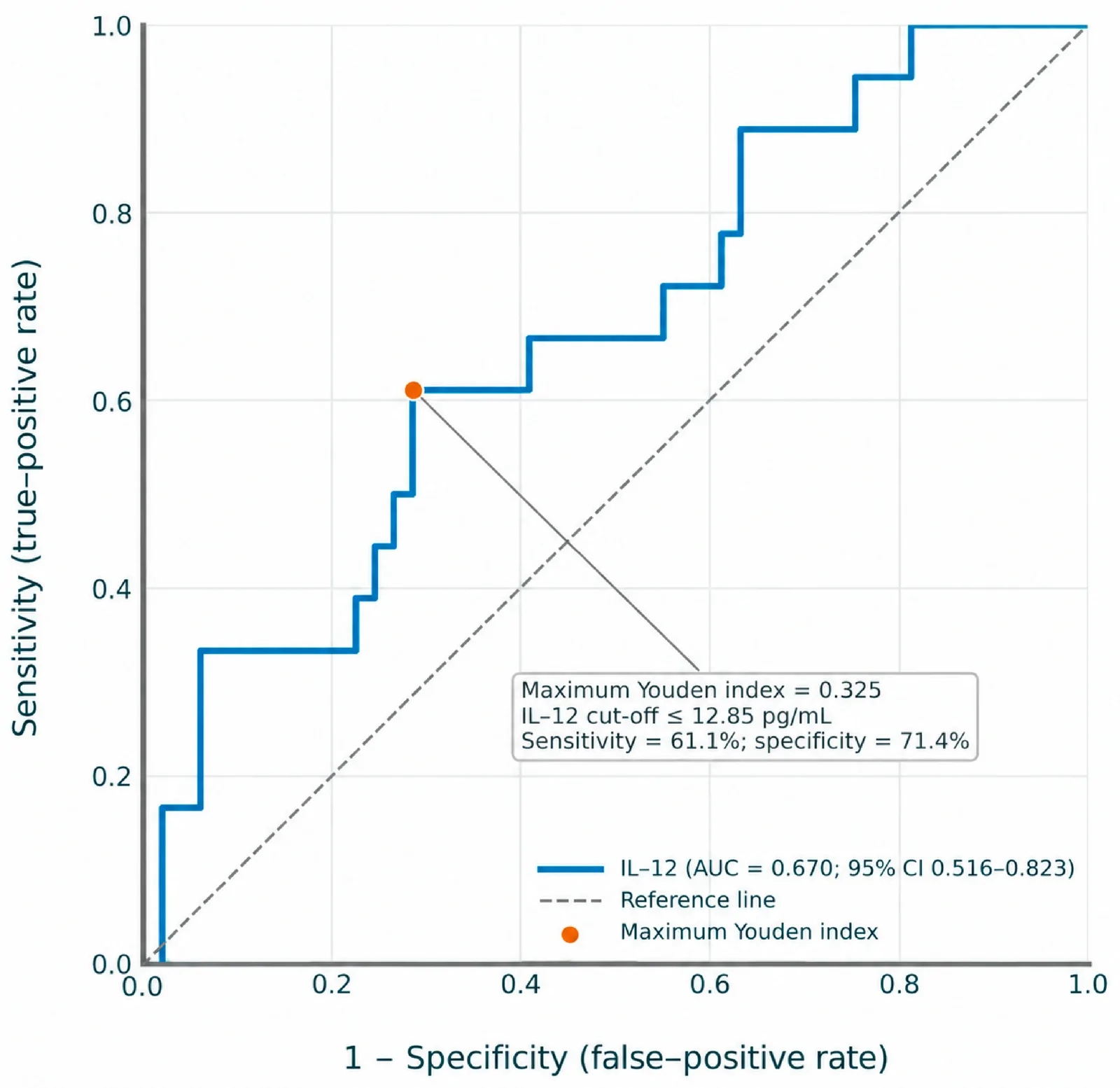
ROC for plasma IL-12 in the discrimination of AAE from non-AAE participants. The analysis included all study participants: patients with AAE (*n* = 18) and the rest of the cohort (*n* = 49), comprising patients with NAE (*n* = 33) and HC (*n* = 16). The area under the curve was 0.670 (95% CI, 0.516–0.823), indicating modest discriminative ability of plasma IL-12 for distinguishing participants with AAE from the rest of our cohort.

Since the correlation between IL-12 levels and the possibility of AAE is inverse, we generated the ROC curves for the inverse variable to IL-12 levels (Figure 2).

The exploratory Youden-index cut-off for plasma IL-12 was 12.85 pg/mL. This threshold yielded a sensitivity of 61.1% and a specificity of 71.4%. Thus, 61.1% of patients with autoimmune epilepsy had IL-12 levels below 12.85 pg/mL, whereas 71.4% of patients without autoimmune epilepsy had IL-12 levels above this threshold and were correctly classified as negative. This threshold was derived and evaluated in the same small cohort and is not intended for clinical use.

**Figure 3:**
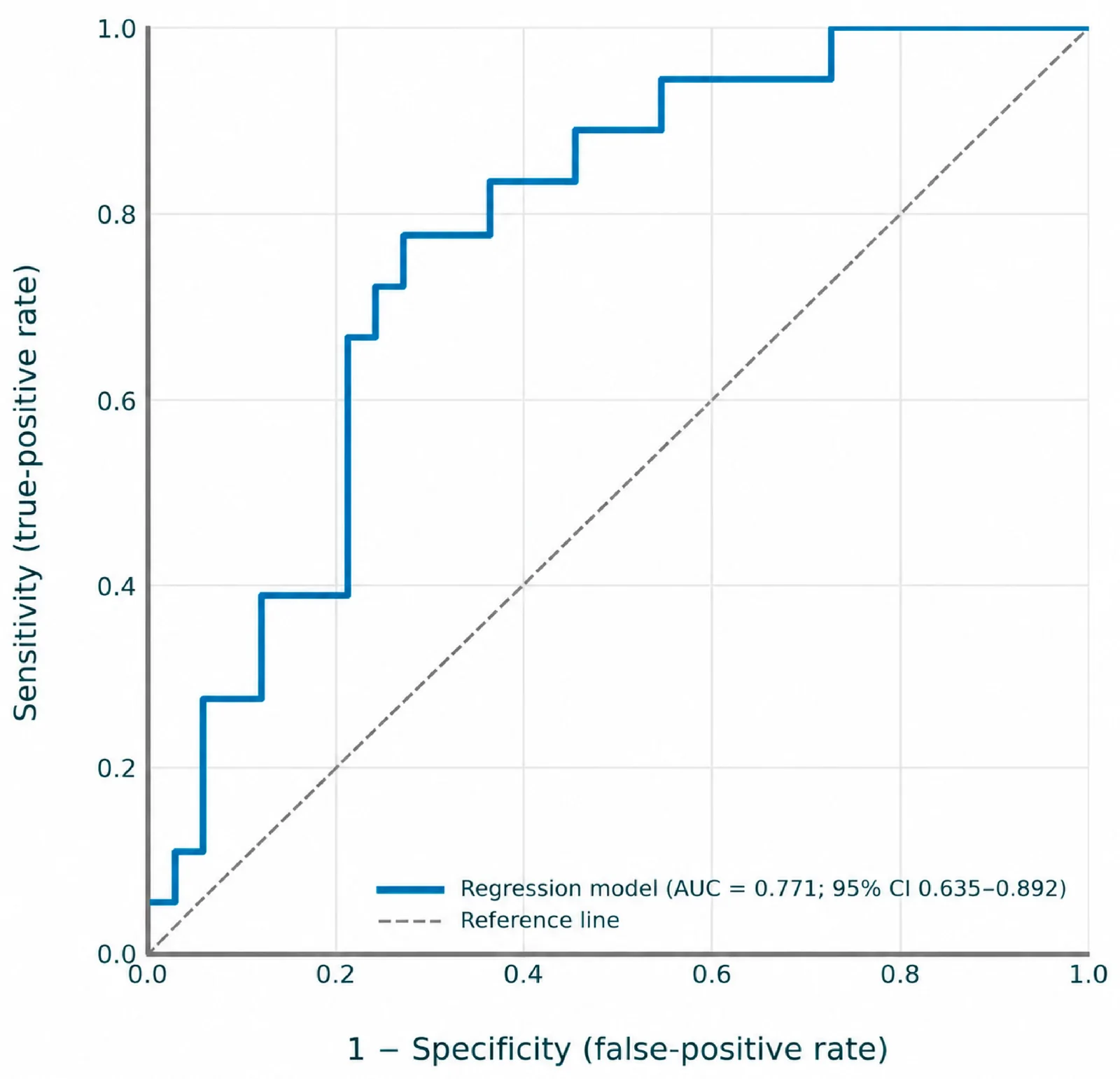
ROC for the exploratory multivariable logistic regression model distinguishing AAE from NAE. The analysis included patients with epilepsy only: AAE (*n* = 18) and NAE (*n* = 33). The model included plasma IL-12 concentration, DRE status, and multifocal IEDs. The apparent in-sample AUC was 0.771 (95% CI, 0.635–0.892).

Next, we generated the ROC curve for the multivariable regression model (Figure 3).

The discriminative performance of the multivariable model was evaluated among patients with epilepsy only, using ROC analysis based on logistic regression-predicted probabilities for distinguishing AAE from NAE. HC were excluded because DRE status and EEG variables were not applicable to this group. The model showed an apparent in-sample AUC of 0.771 (95% CI, 0.635–0.892). This estimate should be interpreted cautiously because the model was developed and evaluated in the same small cohort, without internal resampling or external validation. The model had a Nagelkerke’s *R*^2^ of 0.289 and an apparent classification accuracy of 70.6%.

### 3.5. Exploratory Descriptive Analysis of Immunomodulatory Treatment Exposure

Among the 18 patients with AAE, six had received immunomodulatory therapy at some point during their clinical course, whereas 12 had no documented immunomodulatory treatment. Plasma IL-12 concentration was descriptively lower in the treatment-exposed group than in the non-exposed group (median 10.76 [IQR 7.44–18.19] vs. 12.15 [7.40–39.33] pg/mL, respectively; Mann–Whitney *U* = 26.0, nominal *p* = 0.385; Table S2 and Figure S3). Of the six treatment-exposed participants, samples were collected after immunotherapy in two and before immunotherapy in four. Therefore, this comparison is descriptive and cannot distinguish treatment-related from disease-related differences.

## 4. Discussion

Apart from the nominal group difference in plasma IL-12, which we will discuss later, the remaining cytokines, chemokines, endothelial markers, and damage-associated molecules we included in our study did not show statistically significant between-group differences. This result does not support the presence of a broad and uniform systemic inflammatory signature in this chronic AAE cohort. This is the main result of our study. However, it should not be interpreted as evidence against immune involvement in chronic AAE. Cytokine networks are dynamic, may differ according to disease stage and antibody-associated phenotype, and may be more strongly influenced by recent seizures, treatment exposure, and pre-analytical conditions than by diagnostic category alone [28]. The lack of significant differences in IL-1β, TNF-α, IL-17A, HMGB1, TLR4, adhesion molecules, and chemokines should also be interpreted in the context of limited statistical power and multiple testing. Several of these biomarkers have been associated in prior epilepsy studies with drug resistance, seizure severity, acute seizures, or postictal changes rather than specifically with autoimmune etiology. Thus, their absence as discriminatory markers in the present cohort may reflect the distinction between chronic etiological classification and transient seizure-related inflammatory activity.

The only nominally significant finding was that plasma IL-12 levels were lower in chronic AAE than in NAE and HC. However, this nominal association did not remain significant after false discovery rate correction across the biomarker panel and should therefore be interpreted as hypothesis-generating rather than confirmatory.

Regarding the possible biological basis for this association, it may reflect differences in peripheral immune-cell composition, immunoregulatory feedback, immunotherapy exposure, disease stage, or a dissociation between peripheral and CNS immune activity, rather than a causal role for IL-12. In any case, among the six patients exposed to immunomodulatory therapy, blood sampling preceded treatment in four and occurred after treatment in two (Cases 10 and 18). Therefore, treatment-related effects on plasma biomarkers cannot be excluded, although they would have directly affected only a small subset of the AAE cohort.

The nominal IL-12 pattern observed in this cohort may differ from cytokine profiles reported in active autoimmune encephalitis; however, this comparison remains speculative. IL-12 alone showed modest apparent discrimination (AUC 0.670; 95% CI, 0.524–0.815) and should not be considered a stand-alone diagnostic biomarker. In addition, because both the NAE and AAE groups were clinically and immunologically heterogeneous, the IL-12 association should not be interpreted as a uniform signature of autoimmune or non-autoimmune epilepsy.

Previous literature indicates that IL-12 has multiple roles in neuroinflammation. It has traditionally been regarded as a pro-inflammatory cytokine that promotes type 1 immune responses, supports Th1-cell differentiation and expansion, stimulates the production of IFN-γ and GM-CSF, and enhances NK- and CD8-mediated cytotoxicity [29]. Paradoxically, IL-12 has also been shown in autoimmune diseases, including those affecting the CNS, to exert neuroprotective effects by attenuating immunopathology driven by excessive cellular immune responses. This highlights the complex and context-dependent role of this cytokine [30]. The underlying mechanism behind these opposing properties and the cellular actors involved remains poorly understood.

In the context of an autoimmune disease, a pro-inflammatory dysregulation of the system would be expected, either in the form of an increase in IL-12/Th1 signaling, or through downregulation mechanisms as an adaptive response to limit tissue damage, as has been described in animal models with chronic alteration of IL-12/IL-12R signaling [30,31]. Notably, because IL-12 was the only marker with a nominal between-group difference and no parallel changes were observed in other measured Th1-related cytokines, our findings do not support dysregulation of the IL-12/Th1 pathway in these patients.

Experimental in vitro and animal studies indicate that IL-12 production and IL-12/IL-12R signaling are subject to context-dependent immunoregulatory control; however, the relevance of these mechanisms to chronic AAE remains uncertain [32,33,34].

This pattern contrasts with the acute pro-inflammatory response described in other autoimmune conditions and is consistent with the broader hypothesis of a decoupling between central neuroinflammation and selected systemic pro-inflammatory pathways in advanced disease stages.

Evidence suggests that chronic autoimmune diseases may reshape cytokine networks, potentially leading to relative downregulation of IL-12. For example, in systemic lupus erythematosus (SLE), long-term reconfiguration of the IL-12/IL-23 axis has been described, with the IL-23/Th17 pathway becoming predominant in chronic inflammation and organ damage, while classical Th1 signaling appears attenuated [35]. In the same way, our data showing relatively lower plasma levels of IL-12 in AAE could reflect a similar change in balance between cytokine axes rather than an absence of neuroinflammation, suggesting that the peripheral inflammatory association in AAE could be sustained by immune circuits partially decoupled from systemic IL-12 signaling.

A previous Japanese multicenter study, also case–control, included 23 patients with cases of AAE with positive antibodies (*n* = 13) versus seronegative AAEs (*n* = 10) [36]. Our result differs from the cytokine profile reported by this study in antibody-positive autoimmune epilepsy, in which serum IL-12, IL-23, IL-6, IL-17α, and IFN-γ and CSF IL-6 and IL-17α were elevated. Several differences between the cohorts may be relevant. Hara et al. focused on active antibody-positive disease and described a peripheral and intrathecal immune profile associated with plasmablast expansion and circulating T-follicular helper-cell subsets. In contrast, our cohort predominantly represents chronic epilepsy evaluated in a tertiary epilepsy setting, includes antibody-positive and seronegative AAE and encompasses diverse antibody and clinical phenotypes.

Accordingly, the two studies may capture different biological states. So, acute or clinically active antibody-mediated disease may be associated with increased circulating and intrathecal cytokine activity, whereas chronic disease, longstanding DRE, treatment exposure, or compartmentalization of immune activity may yield a different peripheral profile. This interpretation remains speculative because our cross-sectional design did not include longitudinal sampling or systematic assessment of disease activity at the time of sampling.

An important interpretative limitation is that plasma cytokines are peripheral circulating measures and cannot be considered direct surrogates of neuroinflammation within the CNS. Their concentrations may be influenced by immune-cell activity and by systemic metabolic, vascular, endocrine, hepatic, renal, infectious, and treatment-related factors. Therefore, the lower plasma IL-12 concentration observed in AAE should not be interpreted as evidence that IL-12 signaling is reduced within the brain or CSF compartment.

Conversely, the absence of significant differences in the available CSF biomarkers does not demonstrate the absence of intrathecal inflammation. The CSF analyses were limited to a restricted biomarker set and were underpowered for robust subgroup inference. CNS inflammation in AAE may also be compartmentalized, temporally dynamic, or driven by immune-cell populations and mechanisms not captured by the measured soluble markers. Future studies should incorporate paired plasma and CSF sampling, albumin quotient or other measures of blood–brain barrier integrity, systematic clinical activity assessment, and longitudinal measurements before and after immunotherapy.

Treatment exposure and seizure-related factors are additional potential sources of heterogeneity, including an impact on plasma IL-12 levels [37]. A proportion of patients with AAE had received intravenous immunoglobulin, rituximab, azathioprine, or combinations of these therapies [38]. As can be seen in Table 2, only in two cases (cases 10 and 18) had the patients received immunomodulatory treatment before the sample was collected for analysis. Therefore, only in these cases could the determinations have altered the results. Similarly, antiseizure medications may influence inflammatory pathways, and seizure burden, time since the last seizure, and recent seizure clustering may affect peripheral biomarker concentrations [28].

Although seizure frequency, drug refractoriness, and antiseizure medication exposure were clinically characterized, the study was not powered to model all treatment- and seizure-related covariates simultaneously. These factors should be prospectively standardized in future work, including documentation of interval from the last seizure, seizure clustering, treatment timing, type and cumulative exposure to immunotherapy, and longitudinal sampling before and after therapeutic interventions.

The multivariable model was evaluated only among patients with epilepsy, thereby addressing the clinically relevant distinction between AAE and NAE. HC were included in descriptive biomarker comparisons but excluded from the multivariable model because drug resistance and EEG variables are not applicable to individuals without epilepsy. The observed AUC reflects apparent in-sample discrimination and should not be interpreted as evidence of validated diagnostic performance. This multivariate analysis shows that incorporating clinical variables, such as DRE status and EEG findings, resulted in a higher apparent AUC than IL-12 alone for discrimination between AAE and NAE. This apparent improvement requires confirmation in an independent cohort. The model achieved an AUC of 0.771 (95% CI, 0.635–0.892), indicating acceptable discrimination of AAE from NAE. If validated in independent cohorts, this index may warrant prospective evaluation as part of a multivariable research framework but it is not suitable for clinical decision-making based on the present data.

This study has several limitations. First, the small sample size, particularly within the AAE subgroup, reduces statistical precision and limits the ability to detect modest between-group differences. Consequently, these findings should not be interpreted as establishing a diagnostic biomarker or a clinically applicable cut-off. Second, plasma cytokines are peripheral immune measures and do not establish CNS-specific inflammatory activity or IL-12 signaling. Third, the NAE group combined structural and non-lesional epilepsies, while the AAE group included neuronal surface antibody-associated, intracellular/onconeural antibody-associated, and APE2-positive seronegative cases. This heterogeneity may have diluted subgroup-specific cytokine patterns and limits extrapolation of the nominal IL-12 association to individual antibody-defined syndromes. Nevertheless, despite this heterogeneity, these conditions share a common feature: immune-system activation. We therefore considered that they might share biological processes not present in other epilepsy types, such as StrE or NLE. Our study aimed to explore whether these shared immune-related processes could be reflected in a peripheral circulating biomarker profile, even in the absence of other established biomarkers. Fourth, the AAE group comprised antibody-associated and seronegative cases with potentially distinct immunopathogenic mechanisms; hence, the observed plasma IL-12 association should not be extrapolated to individual antibody-defined syndromes. Finally, the cross-sectional design and reliance on plasma measurements, with NfL assessed only in CSF, preclude conclusions about temporal dynamics, including acute versus chronic phases or changes before and after immunotherapy. Overall, peripheral biomarkers may not fully reflect the central neuroinflammatory milieu, and these results should therefore be considered exploratory and hypothesis-generating.

## 5. Conclusions

Overall, our findings do not support a universal systemic inflammatory signature in chronic AAE or a CNS-specific role for IL-12. Rather, lower plasma IL-12 emerged as a preliminary peripheral immune association in this clinically heterogeneous cohort. No plasma biomarker remained significant after false discovery rate correction; however, IL-12 showed the strongest nominal signal and warrants prospective validation in larger, treatment-stratified cohorts.

This observation differs from the pro-inflammatory cytokine profile reported in active antibody-positive autoimmune epilepsy and may reflect differences in disease stage, immunotherapy exposure, seizure burden, immune compartmentalization, or residual confounding. Future prospective studies should recruit larger and etiologically stratified cohorts, obtain paired plasma and CSF samples at standardized times, document the interval from the last seizure and immunotherapy, measure relevant systemic confounders, and validate any multivariable model in an independent dataset.

## Figures and Tables

**Table 1:** Clinical and demographic characteristics of the cohort. IED: Interictal Epileptiform Discharges.

		HC (*n* = 16)	StrE (*n* = 11)	NLE (*n* = 22)	AAE (*n* = 18)	*p*
Sex (%)	Male	31.25 (5/16)	18.18 (2/11)	54.54 (12/22)	38.88 (7/18)	0.199
	Female	68.75 (11/16)	81.81 (9/11)	45.45 (10/22)	61.11 (11/18)	
Age, mean ± SD (years)		47.44 (19.94)	55.31 (12.82)	50.62 (15.33)	55.07 (19.94)	0.435
Age at epilepsy onset, mean ± SD (years)		N/A	24.19 (9.09)	29.73 (19.47)	36.65 (17.68)	0.026
Epilepsy duration, mean ± SD (years)		N/A	30.18 (11,68)	20.32 (14.85)	17.47 (13,13)	0.321
Type of seizures (%)	Focal seizures	N/A	54.54 (6/11)	59.09 (13/22)	72.22 (13/18)	<0.001
	Focal and bilateral tonic–clonic seizures	N/A	45.45 (5/11)	40.90 (9/22)	27.77 (5/18)	
EEG findings (%)	Focal IED	N/A	63.63 (7/11)	59.09 (13/22)	55.55 (10/18)	<0.001
	Multifocal IED	N/A	9.09 (1/11)	18.18 (4/22)	27.77 (5/18)	
	No IED	N/A	27.27 (3/11)	22.27 (5/22)	16.66 (3/18)	
Etiology (%)	Hippocampal sclerosis	N/A	63.63 (7/11)	0 (0/22)	33.33 (6/18)	<0.001
	Focal cortical dysplasia	N/A	18.18 (2/11)	0 (0/22)	11.11 (2/18)	
	Gliotic lesion	N/A	9.09 (1/11)	0 (0/22)	0 (0/18)	
	Encephalocele	N/A	9.09 (1/11)	0 (0/22)	0 (0/18)	
	Non lesional	N/A	0 (0/11)	100 (22/22)	55.55 (10/18)	
DRE status (%)	Yes	N/A	90.91 (10/11)	50.00 (11/22)	94.44 (17/18)	0.002
	No	N/A	9.09 (1/11)	50.00 (11/22)	5.55 (1/18)	
Frequency of seizures (%)	Daily	N/A	27.27 (3/11)	4.54 (1/22)	27.77 (5/18)	<0.001
	Weekly	N/A	18.18 (2/11)	31.81 (7/22)	33.33 (6/18)	
	Monthly	N/A	27.27 (3/11)	4.54 (1/22)	22.22 (4/18)	
	Annuals	N/A	27.27 (3/11)	13.63 (3/22)	11.11 (2/18)	
	Free	N/A	0 (0/11)	45.45 (10/22)	5.55 (1/18)	
APE2 Score		0	0.19 (0.40)	1.85 (1.14)	4.94 (2.07)	<0.001
Number of concomitant ASMs, mean ± SD		N/A	2.45 (1.03)	2.23 (0.92)	2.28 (1.07)	0.829
Mechanisms of action of the ASM	Sodium channel blockers	N/A	81.81 (9/11)	86.36 (19/22)	83.33 (15/18)	0.935
	GABAergic ASMs	N/A	27.27 (3/11)	27.27 (6/22)	50 (9/18)	0.268
	SV2A	N/A	54.54 (6/11)	36.36 (8/22)	55.55 (10/18)	0.411

**Table 2:** Characteristics of the AAE cohort. IT: Immunomodulatory Treatment.

Case	Sex	Age (Years)	DRE	Frequency of Seizures	Autoantibodies	MRI Findings	EEG Findings	IT	Timing of Sample Collection Relative to IT/Clinical Response	APE2 Score
Case 1	Female	41.11	No	Seizure-Free	Anti-NMDAR in CSF	HS	No IED	None	Not applicable	5
Case 2	Male	59.55	Yes	Weekly	Anti-Ampa in serum and CSF	Normal	Focal IED	None	Not applicable	2
Case 3	Female	73.79	Yes	Weekly	Anti-LGI1 serum	Normal	Focal IED	None	Not applicable	4
Case 4	Female	40.98	Yes	Weekly	Anti-LGI1 serum	Normal	Focal IED	IgIV	Sample collected before IT/Good response	5
Case 5	Male	61.79	Yes	Yearly	Anti-GAD65 serum	HS	Focal IED	None	Not applicable	3
Case 6	Female	54.86	Yes	Daily	Anti-NMDAR in serum	Normal	Focal IED	None	Not applicable	3
Case 7	Female	42.98	Yes	Daily	Anti-Recoverin serum	DCF	Multifocal IED	None	Not applicable	2
Case 8	Female	49.70	Yes	Monthly	No	HS	Focal IED	IgIV	Sample collected before IT/Good response	7
Case 9	Male	68.35	Yes	Weekly	No	DCF	Focal IED	None	Not applicable	8
Case 10	Female	33.56	Yes	Daily	Anti-GAD65 serum	HS	Multifocal IED	IgIV	Sample collected after IT/Poor response	9
Case 11	Female	58.89	Yes	Daily	Anti-GAD65 serum	Normal	Focal IED	IgIV/Rituximab/Azathioprine	Sample collected before IT/Good response	4
Case 12	Female	54.33	Yes	Daily	Anti-AMPA/Anti-GABAb serum	HS	Focal IED	IgIV	Sample collected before IT/Good response	7
Case 13	Male	78.33	Yes	Monthly	Anti-CV2 serum	Normal	Multifocal IED	None	Not applicable	4
Case 14	Female	26.57	Yes	Monthly	Anti-NMDAR serum	Normal	Multifocal IED	None	Not applicable	7
Case 15	Female	56.57	Yes	Weekly	No	Normal	No IED	None	Not applicable	6
Case 16	Male	67.37	Yes	Monthly	Anti-SOX and Anti-YO in serum	Normal	Focal IED	None	Not applicable	4
Case 17	Male	56.62	Yes	Yearly	Anti-Ampa in CSF and serum	Normal	Multifocal IED	None	Not applicable	3
Case 18	Male	65.95	Yes	Weekly	LGI1 serum	HS	No IED	IgIV/Rituximab	Sample collected after IT/Poor response	6

**Table 3:** Comparison of biomarker levels by group. SD: Standard deviation. IQR: Interquartile range. All determinations are expressed in pg/mL. Overall group comparisons were performed using one-way ANOVA or Kruskal–Wallis test, as appropriate.

	Mean (SD)			Median (IQR)			*p*
	HC (*n* = 16)	AAE (*n* = 18)	NAE (*n* = 33)	HC (*n* = 16)	AAE (*n* = 18)	NAE (*n* = 33)	
Plasma biomarkers							
MIP-1α (CCL3)	2.37 (3.16)	5.24 (7.41)	4.16 (8.91)	1.12 (2.00)	2.06 (45.21)	1.58 (2.03)	0.201
IL-1β	9.82 (8.51)	5.14 (3.06)	6.98 (4.37)	6.42 (8.53)	3.98 (4.85)	5.85 (6.61)	0.124
IL-4	12.37 (9.42)	7.11 (2.55)	8.23 (4.73)	9.11 (7.90)	7.20 (3.77)	9.45 (5.35)	0.073
IP-10 (CXCL10)	2.28 (1.57)	3.73 (2.07)	3.04 (2.07)	1.89 (2.11)	3.69 (4.18)	2.09 (3.44)	0.101
IL8 (CXCL8)	1.40 (0.24)	1.14 (0.64)	1.24 (0.58)	1.40 (0.41)	1.23 (0.84)	1.21 (0.37)	0.370
IL-10	1.97 (1.42)	1.09 (0.70)	1.47 (0.90)	1.55 (1.65)	0.78 (1.15)	1.26 (1.26)	0.054
IL-12	40.60 (29.96)	20.40 (16.39)	29.37 (21.64)	39.53 (37.46)	11.14 (31.17)	29.92 (32.53)	0.038
IL-13	9.42 (5.65)	10.17 (5.74)	8.39 (4.39)	6.08 (7.15)	9.29 (9.21)	7.61 (6.74)	0.454
IL-17α	6.94 (3.34)	7.17 (3.10)	6.94 (2.76)	6.19 (4.94)	6.22 (4.66)	6.19 (2.20)	0.963
IL-33	4.73 (2.85)	5.11 (3.12)	3.73 (2.11)	3.90 (4.06)	4.19 (5.21)	3.26 (1.73)	0.197
IFN-γ	2.87 (3.91)	7.10 (5.76)	5.15 (5.64)	1.04 (1.45)	6.97 (10.73)	1.20 (6.44)	0.134
GM-CSF	54.20 (31.67)	30.24 (29.05)	45.44 (49.57)	48.88 (38.58)	20.29 (40.96)	37.63 (47.35)	0.065
TNF-α	13.81 (6.24)	16.34 (7.68)	14.97 (7.42)	12.76 (9.02)	15.52 (15.50)	13.87 (6.23)	0.553
MIP1-β	11.96 (4.60)	12.69 (4.63)	14.56 (12.31)	10.50 (8.63)	10.62 (7.09)	11.27 (6.06)	0.594
IFN-α	1.03 (0.84)	1.34 (0.82)	1.23 (0.72)	0.70 (1.28)	1.14 (1.49)	1.09 (0.93)	0.534
MCP-1	10.34 (6.08)	14.14 (6.60)	15.77 (10.38)	9.94 (7.93)	13.55 (12.29)	12.06 (16.33)	0.126
P-selectin	16,005.10 (39,258.50)	19,858.43 (19,662.15)	23,563.15 (48,252.63)	10,906.98 (13,203.94)	16,351.75 (13,070.51)	14,394.32 (18,134.41)	0.840
IL-1-α	0.57 (0.37)	0.42 (0.18)	0.71 (1.23)	0.48 (0.52)	0.42 (0.61)	0.45 (0.34)	0.613
ICAM-1	35,193.99 (118,445.28)	344,649.52 (996,337.64)	237,822.79 (784,197.36)	49,416.32 (100,146.63)	76,030.69 (119,436.72)	47,132.88 (106,876.74)	0.080
E-selectin	5442.02 (3718.05)	7192.51 (4002.34)	6043.64 (4109.92)	6118.48 (5971.21)	5986.57 (6660.60)	5546.79 (7542.21)	0.424
TLR4 (PL)	2323.72 (1404.43)	2893.76 (2365.11)	2312.81 (2385.67)	1776.14 (2143.75)	1947.53 (2484.17)	1695.68 (1720.56)	0.635
HMGB1 (PL)	9919.10 (11,223.93)	6688.92 (3059.91)	8118.71 (6071.06)	5819.58 (6174.57)	6411.50 (5505.82)	6349.27 (3161.68)	0.957
CSF biomarkers							
TLR4 (CSF)	121.24 (89.12)	171.51 (197.38)	116.88 (89.06)	103.03 (76.69)	11.09 (157.95)	87.56 (78.59)	0.895
HMGB1 (CSF)	212.64 (181.70)	379.39 (333.01)	434.23 (361.67)	204.55 (164.72)	264.11 (268.21)	317.08 (274.99)	0.181
NfL (CSF)	391.61 (317.45)	536.61 (317.74)	670.09 (759.34)	286.31 (346.17)	546.05 (381.90)	414.47 (481.25)	0.145

**Table 4:** Exploratory multivariable logistic regression model distinguishing AAE from NAE. SE = Standard Error.

Predictor	B	SE	OR	OR (95% CI)	Wald	df	*p*-Value
DRE (yes vs. no) Plasma IL-12 Multifocal IED (yes vs. no)	2.578	1.126	13.17	1.45–119.6	5.24	1	0.022
	−0.037	0.019	0.964	0.929–1.000	3.90	1	0.048
	0.564	0.785	1.76	0.38–8.19	0.52	1	0.473

## Data Availability

“Restrictions apply to the datasets”. The datasets presented in this article are not readily available because the data are part of an ongoing study. Requests to access the datasets should be directed to the corresponding author at pedro.serrano.c@uma.es.

## Supplementary Materials

The following supporting information can be downloaded at: https://www.mdpi.com/article/10.3390/biom16091350/s1, Supplementary Section S1. Detailed laboratory methods, confirmatory autoantibody assays, and supplementary analyses; Figure S1. Indirect immunofluorescence staining of anti-GAD antibodies in rat brain tissue. Representative immunofluorescence images showing the characteristic staining pattern of anti-GAD antibodies in the hippocampus (A) and cerebellum (B); Figure S2: STROBE flow diagram; Figure S3. Plasma IL-12 concentration according to immunomodulatory treatment history in patients with autoimmune-associated epilepsy. Individual dots represent participants. Thick vertical bars indicate the interquartile range, and horizontal lines indicate the median. Plasma IL-12 concentrations were descriptively lower among patients with any documented immunomodulatory treatment (*n* = 6) than among those without documented treatment (*n* = 12); however, the difference was not statistically significant (two-sided Mann–Whitney *U* = 26.0, *p* = 0.385). This analysis is exploratory and underpowered, and interpretation is limited by treatment heterogeneity and the fact that samples were obtained after immunotherapy in only two participants; Table S1. Alternative regression model; Table S2. Descriptive comparison of plasma IL-12 according to immunomodulatory treatment exposure in AAE. Values are in pg/mL. No immunomodulatory treatment refers to no documented treatment history. The treated group includes any prior or subsequent treatment; timing is shown separately. IT: Immunomodulatory treatment.

## Abbreviations

The following abbreviations are used in this manuscript:

AAE	Autoimmune-associated epilepsy
AMPAR	α-Amino-3-hydroxy-5-methyl-4-isoxazolepropionic acid receptor
ANOVA	Analysis of variance
APE2	Antibody Prevalence in Epilepsy and Encephalopathy score
ASM	Antiseizure medication
AUC	Area under the curve
CASPR2	Contactin-associated protein-like 2
CBA	Cell-based assay
CCL2	Chemokine (C-C motif) ligand 2
CCL3	Chemokine (C-C motif) ligand 3
CD62E	E-selectin
CD62P	P-selectin
CSF	Cerebrospinal fluid
CV2	Collapsin response mediator protein 5 (CRMP5) paraneoplastic antigen
DAMPs	Damage-associated molecular patterns
DPPX	Dipeptidyl-peptidase-like protein 6
DRE	Drug-resistant epilepsy
EEG	Electroencephalogram
ELISA	Enzyme-linked immunosorbent assay
FCD	Focal cortical dysplasia
GABA-A	Gamma-aminobutyric acid type A receptor
GABA-B/GABABR	Gamma-aminobutyric acid type B receptor
GAD65	Glutamic acid decarboxylase 65 kDa isoform
GM-CSF	Granulocyte-macrophage colony-stimulating factor
HC	Healthy controls
HMGB1	High-mobility group box 1
IED	Interictal epileptiform discharges
IFN-γ	Interferon gamma
IIFT/IIF	Indirect immunofluorescence test
IL-1α	Interleukin-1 alpha
IL-1β	Interleukin-1 beta
IL-4	Interleukin-4
IL-6	Interleukin-6
IL-8	Interleukin-8
IL-10	Interleukin-10
IL-12	Interleukin-12
IL-13	Interleukin-13
IL-17A	Interleukin-17A
IL-18	Interleukin-18
IL-33	Interleukin-33
ILAE	International League Against Epilepsy
IP-10	Interferon gamma-induced protein 10 (CXCL10)
IVIG	Intravenous immunoglobulin
LGI1	Leucine-rich glioma-inactivated 1
MCP-1	Monocyte chemoattractant protein-1 (CCL2)
MRI	Magnetic resonance imaging
NAE	Non-autoimmune epilepsy
NfL	Neurofilament light chain
NLE	Non-lesional epilepsy
NMDAR	N-methyl-D-aspartate receptor
ROC	Receiver operating characteristic
StrE	Structural epilepsy
sTNF-RII	Soluble tumor necrosis factor receptor II
SV2A	Synaptic vesicle glycoprotein 2A
TBA	Tissue-based assay
TLR4	Toll-like receptor four
TNF-α	Tumor necrosis factor alpha
