# Supplementary material for: Peripheral Inflammatory Biomarkers in Chronic Autoimmune-Associated Epilepsy: An Exploratory Case–Control Study
Source: Biomolecules. 2026 Sep 16;16(9):1350. doi: 10.3390/biom16091350 (PMC13604780; doi:10.3390/biom16091350)
Supplement: Supplementary file 1 [file biomolecules-16-01350-s001.zip › biomolecules-4515207-supplementary.pdf]

## Supplementary Section S1. Detailed laboratory methods, confirmatory autoantibody assays, and supplementary analyses.

*Multiplex Assay*

Inflammatory and anti-inflammatory factors were evaluated in plasma samples using a ProcartaPlex™ Human Inflammation Panel, 20-plex kit (catalog number: EPX200-12185-901; Thermo Fisher Scientific Inc., Waltham, MA, USA). Wash Buffer Concentrate (10x) required vortexing and dilution in double-distilled water for 1x solution. Lyophilized multistandards containing the mix of standard proteins were reconstituted to 250 µL with provided Universal Assay Buffer (1x) and used for a 1:4 serial dilution for a standard 7-point curve. The same buffer was used for blank and plasma samples (1:2). As the first step of the assay, the Magnetic Bead solution was vortexed for 30 s and 50 µL was dispensed into each assay well for standards and samples into a 96-well Flat-Bottom Plate inserted into a Handheld Magnetic Plate Washer. Beads settled for 2 min before the liquid was decanted. For washing, 150 µL of wash buffer was added to each well followed by a 30-minute incubation. After decanting, the plate was gently pressed against absorbent paper to remove residual liquid, and the plate was detached from the magnetic plate washer. After that, 50 µL of appropriately diluted standards and experimental samples were added to the designated wells and run in duplicate. The plate was sealed, covered with a black microplate lid to protect from light, and shaken at 500 rpm for 120 minutes at room temperature. Wash steps were performed as described above. After washing (2x), 25 µL of the detection antibody mix was added to each well. The plate was sealed, covered and shaken, as described, for 30 min at room temperature. Following incubation, the plate was washed (2x). Afterward, 50 µL of streptavidin-phycoerythrin (SAPE) solution was added to enable fluorescent labeling of the analyte-bound complexes. The plate was sealed, covered and shaken, as previously, at 500 rpm for 30 min at room temperature. After washing (2x) 120 µL of Reading Buffer was added to each well. The plate was sealed, covered and shaken at 500 rpm for 5 min at room temperature. Then, the cover and seal were removed, and the plate was run for detection using xMAP® technology on the Luminex MAGPIX® instrument (Diasorin S.p.A., Saluggia VC, Italy). Acquisition and analyses were performed using xPONENT® 4.3 software (Diasorin S.p.A., Saluggia VC, Italy).

*ELISA Assay*

Samples were diluted according to the requirements of the corresponding kits. Duplications were made for each sample. Quantification of HMGB1 and TLR4 was performed using an ELISA kit from ElabScience® (Houston, TX, USA), with catalog numbers E-EL-H1554 and E-EL-H1539, respectively. Similarly, samples were tested for sTNFR II using a specific ELISA kit (Human sTNF RII/TNFRSF1B Quantikine® ELISA Kit DRT200; R&D Systems, Inc., Bio-Techne Ltd., Minneapolis, MN, USA).

For HMGB1 and TLR4 ELISA kits, 100 µL of the appropriately diluted standards and samples were added to the microplate wells. The plate was then sealed and incubated for 90 minutes at 37°C. The calibration curve used serial 1:2 dilutions. The dilution of the standards and plasma samples (1:4) was made using the supplied sample dilution buffer. After discarding the liquid without washing, 100 µL of the biotinylated detection antibody working solution was added to each well and incubated for 60 min at 37 °C. After incubation, the liquid was aspirated, and the plate was washed three times by adding 350 µL of wash buffer to each well. After decanting, the plate was gently pressed against absorbent paper to remove residual liquid. Next, 100 µL of the working avidin-HRP conjugate solution was added to each well of the plate, which was then incubated for 30 minutes at 37°C after sealing. Then, the plate was washed five times as previously described and 90 µL of

substrate reagent was added to each well. The plate was sealed and covered from light, then incubated for 15 min at 37°C. Finally, 50 μL of stop solution was added to each well, and the optical density (OD) was measured immediately at 450 nm using an ELISA reader (Lan Technics Model R-100, Hangzhou Allsheng Instruments Co., Ltd., Hangzhou, China).

Regarding the sTNF RII/TNFRSF1B assay, samples required a 10-fold dilution in the provided Calibrator Diluent RD6O, which was also used for the standard curve. A total of 50 μL of Assay Diluent RD1-6 was added to each well. After mixing thoroughly, 200 μL of standard or sample was dispensed per well. The plate was then sealed and incubated at room temperature for 2 hours. Three washing steps were performed as previously described. Next, 200 μL of Human TNF RII Conjugate was added to each well and the plate was sealed and incubated at room temperature for another 2 hours. Following incubation, the plate was washed (3x) and 200 μL of Substrate Solution was added to each well. The plate was then sealed, protected from the light, and incubated for 2 hours at room temperature. Finally, 50 μL of stop solution was added to each well, and the OD was measured as previously described.

The UmanDiagnostics NF-light™ ELISA RUO kit (10-7002, Quanterix Corp., Billerica, MA, USA) was used for NfL measurement in CSF. CSF samples were diluted with equal volume of supplied sample diluent, ensuring a minimum total volume of 210 μL. The standards (8 points, including blank) were reconstituted and diluted following the manufacturer's instructions. Before sample addition, the wells were washed three times by pipetting 300 μL of wash buffer. Then, 100 μL of each standard or sample was added in duplicate. The plate was incubated at room temperature for 1 hour with agitation (800 rpm). After washing, 100 μL of freshly diluted Tracer biotin-labeled antibody (1:50 in sample diluent) was added to each well and incubated at room temperature for 45 min with agitation (800 rpm). The plate was washed again, and 100 μL of freshly diluted Streptavidin-HRP Conjugate (in supplied diluent) was added to each well, followed by 30 min of incubation at room temperature with agitation (800 rpm).

After incubation, the plate was washed, and 100 μL of TMB substrate solution was added to each well. The plate was then protected from the light and incubated for 15 min at room temperature. Finally, 50 μL of stop reagent solution was added to each well, and the OD was measured as previously described.

Samples were diluted according to the requirements of the corresponding kits. All samples were analyzed in duplicate.

Analyses were performed following the manufacturers' standard protocols, with appropriate quality controls to ensure the accuracy and reliability of the results.

*Confirmation methods for detection of Autoantibodies:*

*Tissue-Based Indirect Immunofluorescence*

Rat brains obtained from surplus animals from the University of Málaga animal facility were fixed in 4% paraformaldehyde (PFA), cryosectioned at 10 μm, and stored frozen until use.

Frozen sections were thawed at room temperature for 25 min, surrounded with a hydrophobic barrier (DakoPen), and washed in 1× phosphate-buffered saline (PBS) for 10 min. Sections were then blocked for 1 h at room temperature with 300 μL of blocking solution. Cerebrospinal fluid (CSF) samples were diluted 1:10 and serum samples 1:100 in PBS containing 3% normal goat serum (NGS) and 3% bovine serum albumin (BSA). A volume of 300 μL of diluted sample was applied to each section, followed by overnight incubation at room temperature in a humidified chamber.

The following day, sections were washed three times in 1× PBS for 5 min and incubated for 1 h at room temperature in the dark with goat anti-human IgG (H+L) Alexa Fluor 488 (1:1500) and DAPI (1:200) prepared in blocking solution. Sections were subsequently washed three times in PBS and mounted using FluoPreserve Mowiol mounting medium. Slides were stored at 4 °C for at least 20 min before evaluation.

Fluorescence signals were examined by microscopy and recorded systematically. Tissue-based indirect immunofluorescence was used as a qualitative confirmatory assay for clinically relevant or discordant autoantibody findings.

*Cell-Based Assays*

In-house cell-based assays (CBAs) were performed using transiently transfected HEK293 cells expressing the antigen of interest. Plasmid DNA was obtained by transformation and amplification in Escherichia coli DH5$\alpha$ cells and purified using the PureLink™ HiPure Plasmid DNA Purification Midiprep Kit (Thermo Fisher Scientific) according to the manufacturer's instructions. Purified DNA was quantified using a NanoDrop spectrophotometer and stored at −20 °C.

HEK293 cells were seeded on poly-L-lysine-coated 12 mm coverslips in 24-well plates at approximately 75,000–85,000 cells/well in DMEM supplemented with 10% fetal bovine serum (FBS) and penicillin/streptomycin. Cells were cultured overnight at 37 °C in a humidified 5% $CO_2$ atmosphere.

The following day, cells were transfected with 0.75 µg plasmid DNA per well using Lipofectamine LTX and PLUS Reagent (Thermo Fisher Scientific). Where applicable, 0.15 µg of GFP plasmid was co-transfected as a transfection marker. For NMDAR constructs, medium was supplemented with ketamine at 500 µM during transfection. After 24 h, transfection medium was replaced with fresh DMEM containing 10% FBS and penicillin/streptomycin, and cells were incubated overnight.

For live-cell immunostaining, patient serum and CSF samples were diluted 1:20 and 1:10, respectively, in DMEM–HEPES containing 5% NGS and 1% BSA. Samples were incubated with live cells for 2 h at room temperature in the dark. Cells were washed, fixed with 1% PFA for 15 min, and incubated for 1 h with anti-human IgG Alexa Fluor 555 (1:1200). Coverslips were mounted using Mowiol.

For fixed-cell immunostaining, cells were fixed with 4% PFA for 15 min and blocked/permeabilized for 45 min in PBS containing 0.2% gelatin and 0.1% Triton X-100. CSF samples were diluted 1:10, or 1:5 for NMDAR assays, and serum samples 1:100. Samples were incubated for 1.5 h at room temperature in the dark. For HA-tagged constructs, anti-HA Alexa Fluor 488 antibody (1:200) was included. After washing, anti-human secondary antibody (1:1000) and DAPI (1:1000) were applied for 1 h. Coverslips were mounted with Mowiol, air-dried for at least 1 h, and stored at 4 °C.

Positive control samples and negative serum controls were included in each run. Results were assessed by fluorescence microscopy according to antigen-specific staining patterns and were systematically recorded.

**Table S1.** Alternative regression model.

| | B | Standard error | OR= Exp(B) | OR IC 95% | Wald | gl | Sig. |
|---|---|---|---|---|---|---|---|
| **APE2** | 2,820 | 1,023 | 16,78 | 2,27 – 124,2 | 7,61 | 1 | 0,006 |
| **IL12** | −0,072 | 0,036 | 0,93 | 0,87 – 0,999 | 4,10 | 1 | 0,043 |
| **Multifocal IED** | −1,304 | 1,183 | 0,27 | 0,03 – 2,25 | 1,22 | 1 | 0,270 |

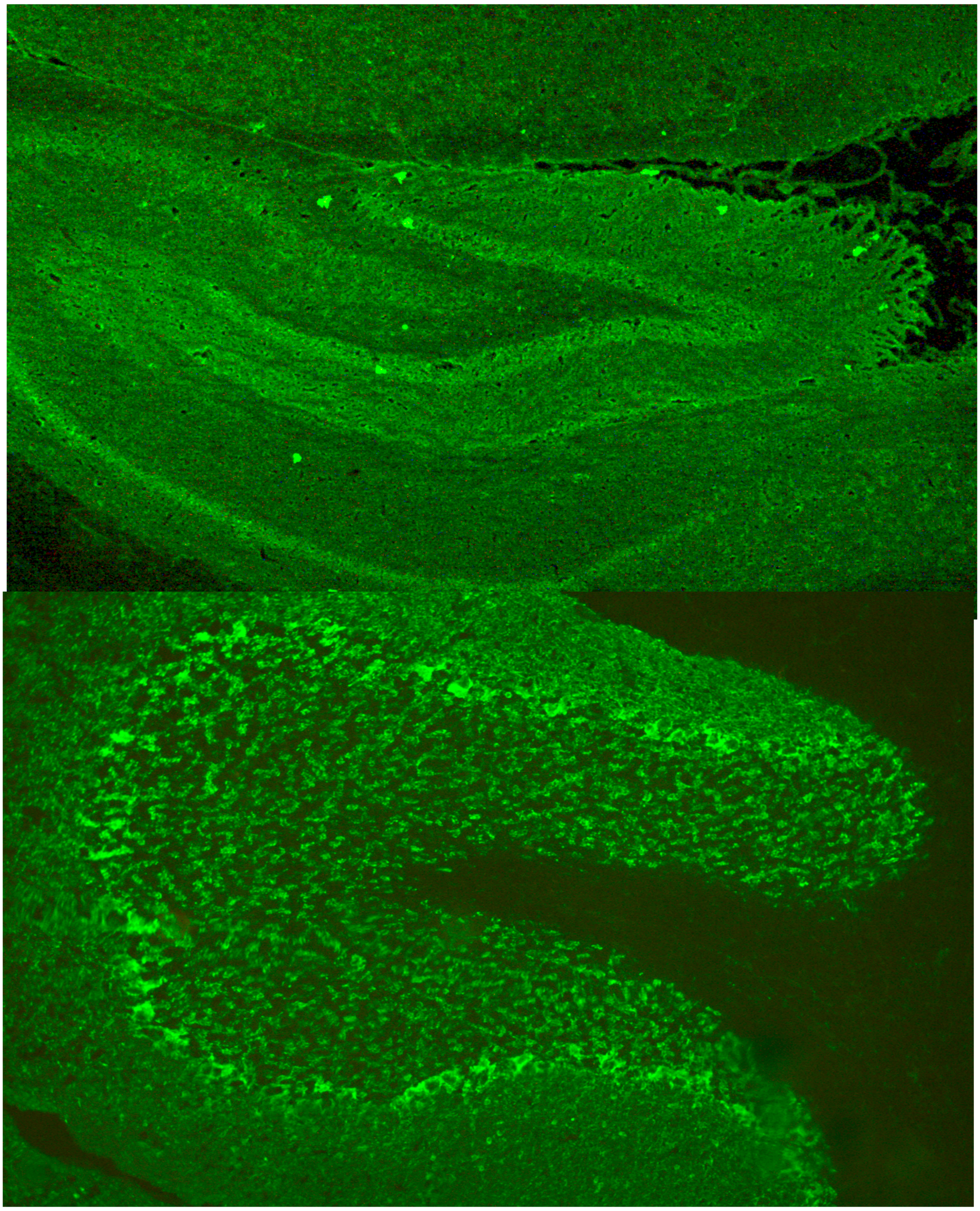

**Figure S1.** Indirect immunofluorescence staining of anti-GAD antibodies in rat brain tissue. Representative immunofluorescence images showing the characteristic staining pattern of anti-GAD antibodies in the hippocampus (A) and cerebellum (B).

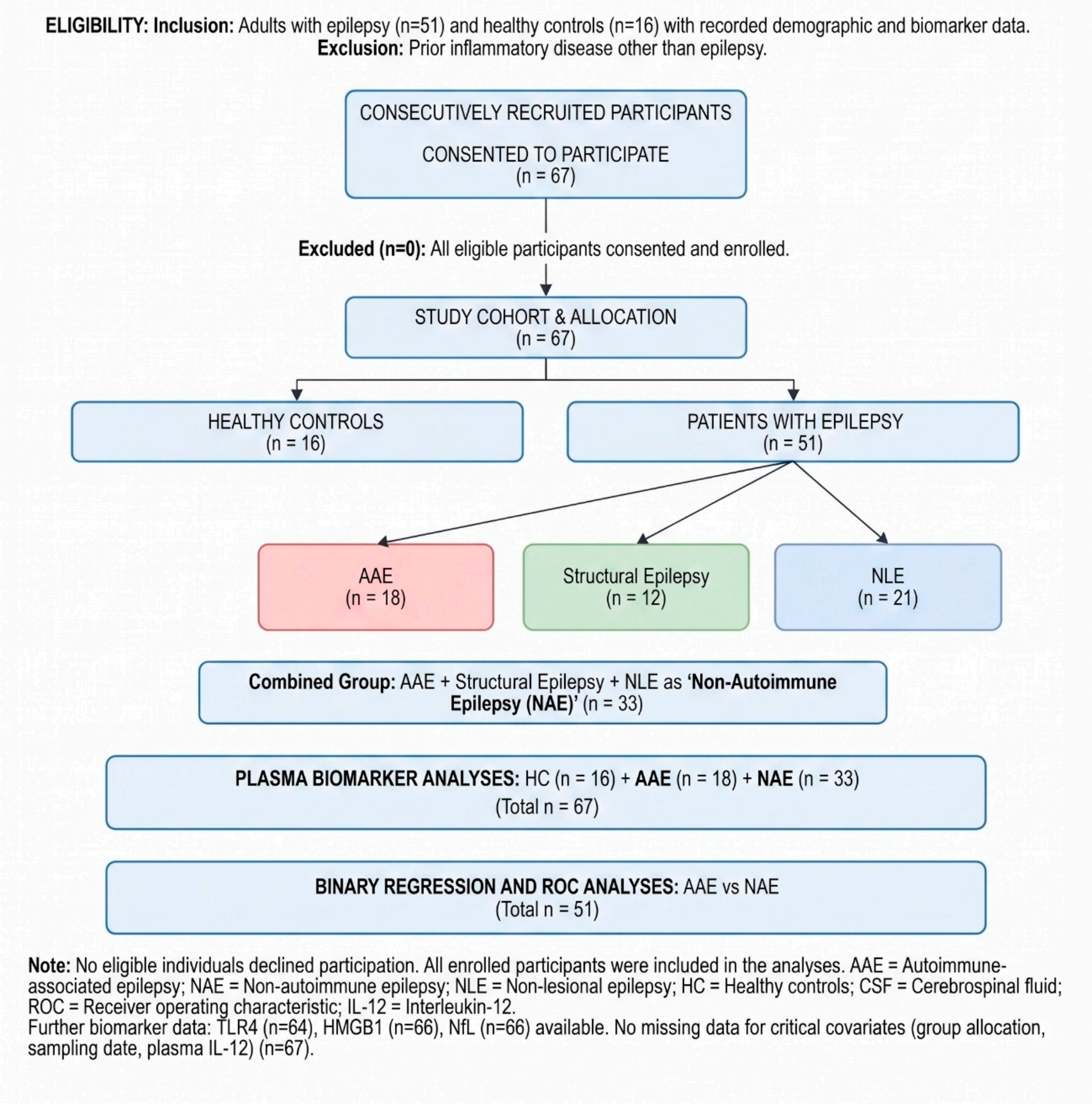


**Figure S2.** STROBE flow diagram.

**Table S2.** Descriptive comparison of plasma IL-12 according to immunomodulatory treatment exposure in AAE. Values are in pg/mL. No immunomodulatory treatment refers to no documented treatment history. The treated group includes any prior or subsequent treatment; timing is shown separately. IT: Immunomodulatory treatment.

| Group | n | Mean | SD | Median | Q1 | Q3 | Minimum | Maximum |
|---|---|---|---|---|---|---|---|---|
| No IT | 12 | 22,93 | 17,99 | 12,15 | 7,4 | 39,33 | 5,6 | 53,39 |
| Any IT | 6 | 15,35 | 12,47 | 10,76 | 7,44 | 18,19 | 5,29 | 38,28 |
| Sample collected after IT | 2 | 24,48 | 19,52 | 24,48 | 17,58 | 31,38 | 10,68 | 38,28 |
| Sample collected before IT | 4 | 10,78 | 7 | 8,6 | 6,1 | 13,29 | 5,29 | 20,64 |

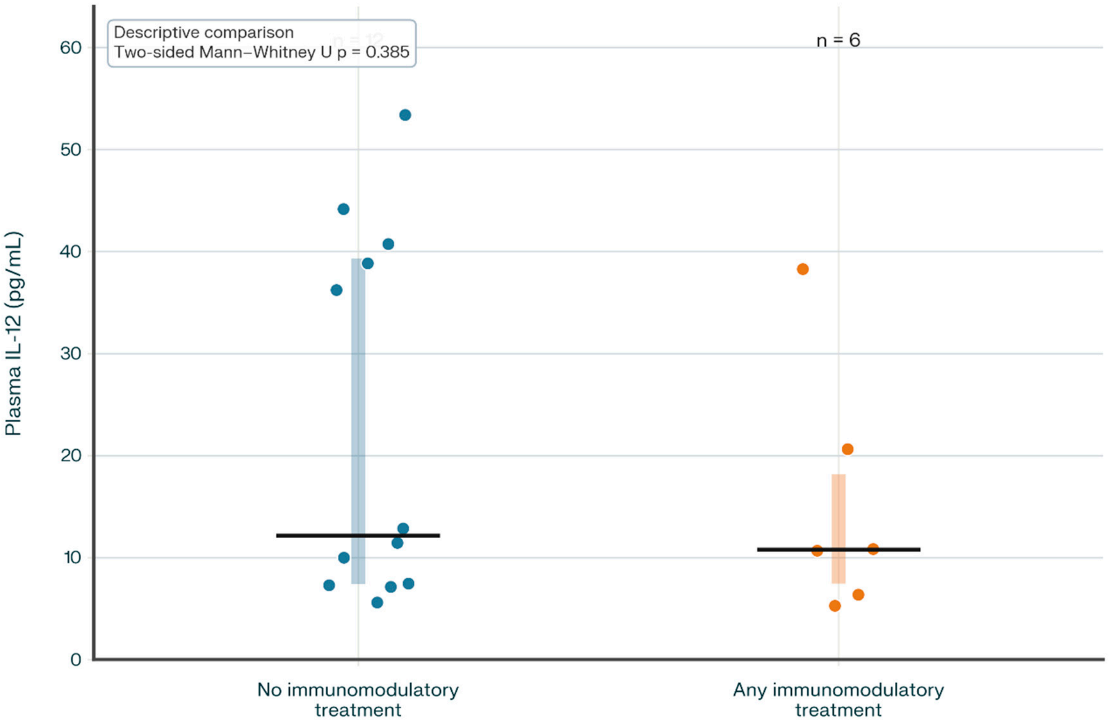


**Figure S3.** Plasma IL-12 concentration according to immunomodulatory treatment history in patients with autoimmune-associated epilepsy. Individual dots represent participants. Thick vertical bars indicate the interquartile range, and horizontal lines indicate the median. Plasma IL-12 concentrations were descriptively lower among patients with any documented immunomodulatory treatment ($n$=6) than among those without documented treatment ($n$=12); however, the difference was not statistically significant (two-sided Mann–Whitney $U$=26.0, $p$=0.385). This analysis is exploratory and underpowered, and interpretation is limited by treatment heterogeneity and the fact that samples were obtained after immunotherapy in only two participants.
